# Strangulated Hernia, Etc.

**Published:** 1858-10

**Authors:** William Dickinson

**Affiliations:** Boston, Mass.


ARTICLE II.

### CASE: STRANGULATED HERNIA--ENTERO-EPIPLOCELE--ARTIFICIAL ANUS—FÆCAL FISTULA—CURE.

BY WILLIAM DICKINSON, M. D., BOSTON, MASS.

Mrs. G., forty-eight years of age, married, the mother of one child, of spare figure and short stature; general health good; bowels habitually costive; has had left inguinal hernia for eight years, descending and returning spontaneously, or readily reduced by assuming the recumbent position and slight manipulation; it had, however, never given her pain, or caused much inconvenience.

*March* 12*th*, 1858—*Friday*.—Ate her dinner as usual, consisting, on this day, of brown bread and butter with strong coffee. At 1 o'clock, one hour after eating, was suddenly seized with severe pain at the epigastrium, with some inclination to go to stool; sought relief at the water closet, and while there strained forcibly with the view of encouraging an operation; obtaining no relief, she returned to the house and to her bed. At 2 p.m., she took an ordinary dose of "salts and senna." This she vomited at 3 o'clock, but with no alleviation of the pain. At 4 o'clock I was called; found her still suffering from pain located vaguely in the region of the stomach. Considering it a case of indigestion, I ordered an emetic of ipecac., to be assisted by copious draughts of warm water. After its operation, though but little food was ejected, she expressed herself much relieved. Some degree of pain persisting, ordered ʒj. to be taken each hour till relieved, of a mixture, consisting of:

| | | | | |
|---|---|---|---|---|
| ℞ | Syr. tolutani, | ℥j. | Morph. sulph. | gr. iij. |
| | Aq. menth. vir. | ʒv. | Chloroform, | ʒiij. |

Mix. Fomentations over the seat of pain were also ordered. I then left, desiring to be informed if farther attendance was requisite, but did not again see her till at the hour of operation.

13*th*—*Saturday*.—She accidentally discovered a small tumor in the right inguinal region. Dr. W., in whose family she had formerly been engaged as nurse, was called. She informed him of the existence of this tumor, which, upon being examined, he pronounced to be a hernia. This he attempted to reduce. Most faithful and appropriate efforts by himself and son were made both with and without ether to reduce it till 11½ o'clock p.m. They then left, with directions that, should the emergency arise, I should be summoned; and the request was left at my house, that, should I determine upon an operation, they should be informed, desiring to be present.

14*th*—*Sunday*.—Dr. Gay was called at 11, and found a case of strangulated hernia. No expectation of relief being obtained except from an operation, this was determined upon, and the hour of 2 o'clock was assigned for this purpose. Dr. Gay, assisted by Dr. H. G. Clarke, performed the operation, other gentlemen being present. I then saw her for the first time since the 12th. An incision of about four inches having been made, and the subjacent tissues carefully dissected, the sac was reached, presenting, with its contents, a lobulated tumor. On opening it, two large masses of omentum in a highly congested state were brought to view, and at the base of this mass was a small knuckle of intestine, including about one-half its circumference firmly engaged in the ring. This portion still retained its peculiar shining appearance, but highly congested and presenting a color inclining to purple. The constriction at the ring around both was so tense, that not even the extremity of the little finger could be insinuated between the hernial masses and the ring. Dr. Gay therefore introduced the knife independently, and made the appropriate incision. After which, the intestine and omentum were in succession reduced; the former readily; but a considerable degree of force was requisite to return the omentum, in consequence of adhesions having been contracted between it and the peritoneum at the margin of the ring. The whole being returned, the outer wound was united by the interrupted suture and adhesive plaster, and over these were applied compresses wet with cold water and the usual bandage. Dr. Gay residing at a considerable distance, I was desired to take the subsequent charge of the patient, and ordered a solution of morphia, with directions that it should be taken if severe pain should occur. During the remainder of the day, ensuing night, and indeed the week following, she experienced but little pain, and every aspect of the case appeared of the most favorable character. The external incision seemed disposed to unite by first intention, and did unite at each extremity, and throughout the interval between the upper extremity and the first suture.

On the third day, a slight degree of peritonitis and limited in extent supervened, the abdomen somewhat tympanitic, the pulse never exceeding 84, tongue clean, no headache or other unpleasant symptom. The peritonitis was treated by morphia; the bowels responded kindly to a cathartic of castor oil and lemon juice, assisted by an enema of mist. assafœtida administered on 17th, it being the fourth day after the operation. Morphia afterwards resumed to answer the indications before alluded to. Discharges of varying colors and consistence escaped from the wound, with but little pus at any time. This condition of things continued till the 22d, eight days after the operation.

23*d.*—With surprise and extreme regret I observed a small quantity of fæcal matter engaged in the external wound, near the lower extremity.

24*th.*—A large slough, two and a half inches in length by half an inch broad, was discovered in the same situation; upon removing this, a copious discharge of liquid fæces with flatus followed.

25*th.*—Another slough of nearly the ssme size was removed from the same situation. Through the fistula, fæcal matter and flatus continued to discharge with but little variation, either in quantity or consistence, till the 29th, fifteen days after the operation. The introduction of liquids or solids into the stomach stimulated peristaltic action, and consequently provoked increased discharge. At this time, 29th, without obvious cause, it ceased, and its cessation was followed by a considerable amount of vomiting. To secure an operation throughout the entire intestinal tract, castor oil with lemon juice, aided by enemata of mist. assafœtida, were administered. The enema was duly returned, followed by a full fæcal discharge; the oil, though retained and exciting a considerable amount of peristaltic action, failed to restore the discharge through the fistula, or to remove the intestinal occlusion. The abdomen became tympanitic, but percussion gave little or no pain; vomiting occurred several times during the day; pulse increased in frequency, but diminished in force; countenance depressed; tongue thickly coated.

31*st*—*Wednesday.*—Symptoms less imminent in the morning. Ordered ext. senna fl. in ʒss. doses, with enema of mist. assaf. ℥iv. aq. oj. The senna was vomited and the enema returned with but traces of fæcal matter. In the afternoon abdomen highly tympanitic; much pain in abdomen, and so intense in evening as to require large doses of morph., and even the inhalation of ether and chloroform during a portion of the night, to procure relief.

*April* 1*st*—*Thursday.*—Symptoms all aggravated in severity. Countenance anxious; tongue much coated; pulse quite strong, and abdomen more tumefied.

2*d*—*Friday.*—Appears brighter and converses with stronger voice. The only hope being in the reopening of the fistula, or in the removal of the occlusion, recourse was again had to copious enemata of soap and water, to the extent of six pints. Of this large amount but one and a half pints were returned.

3*d*—*Saturday.*—Tongue dry, dark colored and furrowed, yet but little sensation of thirst; abdomen more tympanitic; pulse 120, and compressible. Champagne, brandy, quinine and nutritious articles of food given. These were retained but in part, vomiting occurring occasionally, and the substance ejected slightly stercoraceous.

It is an interesting circumstance worthy of mention in this connection, that at this time her friends, having abandoned all expectation of her recovery, had ordered her grave clothes and coffin, and an undertaker engaged, and some preliminaries, even; were made for an autopsy; all awaiting only the contingency of her decease.

4*th*—*Sunday.*—Symptoms quite unchanged. Another attempt for a fæcal operation was made by an indefinite quantity of inf. menth. vir. administered per anum, and soon after ext. senna by the mouth. The senna was ejected after the lapse of an hour, and one-third part of the injection was voided, but with no satisfactory results. But little pain experienced; pulse 124, feeble and compressible. In the afternoon erysipelatous blush of lids of left eye observed.

*5th—Monday.*—Erysipelas of the lids now fully established, with tendency to spread down upon the cheek. As if to compensate for this unwelcome complication, discharge through the artificial anus was now happily restored, after a discontinuance of seven days. This continued during the day in considerable quantity, accompanied by the escape of much flatus. Vomiting and all other unfavorable symptoms now wanting. Took ice cream with much relish, and oyster water.

*6th—Tuesday.*—Tympanites almost entirely subsided; erysipelas circumscribed; pulse 132; tongue less dry and rugose. Ordered quiniæ sulph. gr. ij. 3 t. d., with wine; whisky punch; nourishing but unstimulating food. No discharge per anum.

*7th.*—Erysipelas revived and spreading upon the nose; pulse 120; tongue less dry.

At the time of the operation, March 14th, the hernia of the left side was down, but was afterwards readily reduced. It never having caused her inconvenience, its condition was overlooked. This became the seat of inflammation, and on the 16th had attained such a degree that efforts to reduce it were deemed unadvisable. It was treated as other local inflammations by fomentations, poultices, etc., day by day, without very obvious change in its condition. The tumor became circumscribed, and subsequently declared the presence of pus.

*March 28th.*—It was laid open, when it discharged freely of laudable pus. A mass of omentum was visible in the field of the incision; but no sloughs were at any time released. Pus in constantly diminishing quantity was discharged, till it ultimately healed as an ordinary abscess.

*April 7th.*—Erysipelas progressing upon right cheek; still expression of countenance cheerful; sleep of night continuous and refreshing; discharge from both groins continues, fæces from the one and pus from the other. Ordered enemata of strong beef tea, oj.; wine and quinia internally; and a lotion of ferri sulph. ʒj., glycerin, ℥j., aq. ℥ij., by saturating small pieces of cotton cloth, applied to the affected surface.

*April 9th.*—Erysipelas extended to the forehead and there arrested; pulse 100; discharge as on 7th; tympanites wanting.

*April 11th.*—Symptoms assuming an unfavorable appearance; removed a small slough from left lower eyelid; tonic regimen energetically pursued.

*April 12th.*—Pulse rapid and compressible; countenance anxious; conversation laborious; skin of lids of left eye livid, as also the wounds in each groin; in fine, all symptoms decidedly unfavorable. Continue the same treatment as on 11th.

*April 13th.*—Symptoms less aggravated. A collection of pus in left upper eyelid opened, discharging about ʒj.

*April 14th.*—No discharge from lid; tongue moist, and patient in every respect much improved. The sites of the hernia assuming a healthy aspect. The quinia, which had been hitherto continued, being disliked, was suspended for a few days, then resumed, and nutritious injections continued.

*April 15th.*—But little discharge from artificial anus; no fæces since 7th instant; and in addition to her customary food ate a fig, which, in a diagnostic point of view, proved of essential value.

*April 16th.*—Ordered a copious enema of soap suds. In due time this was returned, and there followed, as stated, a discharge of about "one quart" of solid fæces, accompanied by the escape of flatus. This dejection was not preserved for inspection. She was much prostrated by the operation, it being attended by considerable pain. Feeling a desire for farther evacuation on 17th, ordered another similar enema, which was followed by a very full fæcal dejection, being cylindrical in form and of large diameter. This was carefully examined, but no slough was found, as of intestine, but found seeds of the fig eaten on 15th. Thus, to my great gratification and relief, was demonstrated the continuity of useful intestine throughout the entire tract. For eighteen days at least, the last being on March 29th, an obstruction had existed, effectually preventing the normal action of the intestines. From this date her recovery was rapid and uninterrupted. The wound of left groin speedily healed. Through the fæcal fistula passed in small quantity, serum discolored by bile, and traces of pus and flatus. On the 19th, fig seeds were found upon the compress placed over the fæcal fistula. A fig was eaten on the 18th. This again furnished proof of the communication of the sinus with the intestine. Eggs, tomatoes, oranges, constituting a part of her "bill of fare," portions of these were found in her dejections; and three pearls of considerable size were also found, to tell the tale of indulgence in oysters. Morphia was suspended on 22d, though not without a murmur. In a dejection of 23d was found a slough, measuring one inch in length by three-eighths of an inch in width, and on 26th, another of same size and appearance. The latter was examined by the microscope, but found to differ but little from ordinary cellular tissue. In the absence of *satisfactory* reasons to which to attribute the occlusion and its subsequent release, speculation must supply those most plausible.

During the latter part of her confinement, she was visited by a troublesome bed-sore, which, under appropriate treatment, ultimately healed, and she is now, July 30th, perfectly well, happy in her unexpected restoration, and in the assurance of having obtained a radical cure of her recent hernia. That of the left side remains in the same condition as before its capricious manœuvres. For security she wears a double truss.

